# Author Correction: Evidence of an upper ionospheric electric field perturbation correlated with a gamma ray burst

**DOI:** 10.1038/s41467-023-44224-9

**Published:** 2023-12-21

**Authors:** Mirko Piersanti, Pietro Ubertini, Roberto Battiston, Angela Bazzano, Giulia D’Angelo, James G. Rodi, Piero Diego, Zhima Zeren, Roberto Ammendola, Davide Badoni, Simona Bartocci, Stefania Beolè, Igor Bertello, William J. Burger, Donatella Campana, Antonio Cicone, Piero Cipollone, Silvia Coli, Livio Conti, Andrea Contin, Marco Cristoforetti, Fabrizio De Angelis, Cinzia De Donato, Cristian De Santis, Andrea Di Luca, Emiliano Fiorenza, Francesco Maria Follega, Giuseppe Gebbia, Roberto Iuppa, Alessandro Lega, Mauro Lolli, Bruno Martino, Matteo Martucci, Giuseppe Masciantonio, Matteo Mergè, Marco Mese, Alfredo Morbidini, Coralie Neubüser, Francesco Nozzoli, Fabrizio Nuccilli, Alberto Oliva, Giuseppe Osteria, Francesco Palma, Federico Palmonari, Beatrice Panico, Emanuele Papini, Alexandra Parmentier, Stefania Perciballi, Francesco Perfetto, Alessio Perinelli, Piergiorgio Picozza, Michele Pozzato, Gianmaria Rebustini, Dario Recchiuti, Ester Ricci, Marco Ricci, Sergio B. Ricciarini, Andrea Russi, Zuleika Sahnoun, Umberto Savino, Valentina Scotti, Xuhui Shen, Alessandro Sotgiu, Roberta Sparvoli, Silvia Tofani, Nello Vertolli, Veronica Vilona, Vincenzo Vitale, Ugo Zannoni, Simona Zoffoli, Paolo Zuccon

**Affiliations:** 1 Department of Physical and Chemical Sciences, University of L’Aquila, 67100 L’Aquila, Italy; 2 National Institute of Astrophysics, IAPS, Rome, 00133 Italy; 3 Department of Physics, University of Trento, Povo, Italy; 4 TIFPA-INFN, Povo, 38123 Trento, Italy; 5 National Institute of Natural Hazards, Ministry of Emergency Management of China, Beijing, 100085 People’s Republic of China; 6 INFN, University of Rome Tor Vergata, Rome, 00133 Italy; 7 INFN - Sezione di Torino, 10125 Torino, Italy; 8 INFN-Sezione di Napoli, Naples, 80126 Italy; 9 Dipartimento di Ingegneria e Scienze dell’Informazione e Matematica, University of L’Aquila, 67100 L’Aquila, Italy; 10 Uninettuno University, 00186 Rome, Italy; 11 University of Bologna, Bologna, 40127 Italy; 12 INFN - Sezione di Bologna, 40127 Bologna, Italy; 13 Fondazione Bruno Kessler, 38123 Povo, TN Italy; 14 CNR, V. Fosso del Cavaliere 100, 00133 Rome, Italy; 15 Agenzia Spaziale Italia, Rome, 00133 Italy; 16 Università degli Studi di Napoli Federico II, 80126 Naples, Italy; 17 Department of Physics, University of Rome Tor Vergata, Rome, 00133 Italy; 18 INFN-LNF, Frascati, Rome, 00100 Italy; 19 IFAC-CNR, Sesto Fiorentino, Florence 50019 Italy; 20 National Space Science Center, Chinese Academy of Sciences, Beijing, 100190 People’s Republic of China

**Keywords:** Energy storage, Materials for energy and catalysis

Correction to: *Nature Communications* 10.1038/s41467-023-42551-5, published online 14 November 2023

In this article the author name Mauro Lolli was incorrectly written as Marco Lolli. The original article has been corrected.

